# Supplementary material for: Shift-work sleep disorder among health care workers at public hospitals, the case of Sidama national regional state, Ethiopia: A multicenter cross-sectional study
Source: PLoS One. 2022 Jul 8;17(7):e0270480. doi: 10.1371/journal.pone.0270480 (PMC9269933; doi:10.1371/journal.pone.0270480)
Supplement: Attachment — Submitted filename: Response to reviewer.docx [file pone.0270480.s001.docx]

**Authors’ point by point response to the editor and reviewers**

**Title:** Shift-work sleep disorder among health care workers at public Hospitals, the case of Sidama national regional state, Ethiopia: A multicenter cross-sectional study

**Manuscript ID:** PONE-D-21-37990R1

**Dear editor/reviewers.**

We would like to thank you for your constructive and valuable comments and sugestions that would improve the manuscript. We have addressed all comments and suggestions one by one and hence revised the manuscript thoroughly. Our point-by-point responses for each comment and suggestion are described below.

Thank you for considering our manuscript again.

**Responses to the editor’s comments**

**Comment 1:** Please review your reference list to ensure that it is complete and correct. If you have cited papers that have been retracted, please include the rationale for doing so in the manuscript text, or remove these references and replace them with relevant current references. Any changes to the reference list should be mentioned in the rebuttal letter that accompanies your revised manuscript. If you need to cite a retracted article, indicate the article’s retracted status in the References list and also include a citation and full reference for the retraction notice.

**Author’s response: Thank you editor for the comment.** We have revised our manuscript extensively modified the format, spelling error and reference citation for completeness and correct ness and crosschecked for appropriate cittion requirments for each article we have used.

**Response to Reviewers comments**

**Reiviwer #2**

“Good to see the paper with such substantial change. However, the following things should be taken in to consideration.”

**Comment 1:** . It was commented to describe how predictor variables were entered into the logit models (e.g., entry, forward, backward, hierarchical). However, please ignore it because it does not apply to STATA.**.
Authors' response**. **Thank you reviewer for the comment**. To mention the selection technique for varaibles to requriet for the regression model in STATA is not applicable. As per your comments we have removed it. (Line 183-185, Page 9).

**Comment 2:**  On the discussion part, I still recommend you to discuss factors that don’t have an association in the current study but had associations in other previous studies.

**Authors^'^** **response**: **Thank** **you** **reviewer** **for** **the** **comments**. We tried to discuss those variables which were associated with SWSD in many literatures but not in our finding in the manuscript. As per the comments and your recommendation we also incorporated more variables with significant association with SWSD in other literatures but not here and discussed them in detail in the manuscript. The factors and possible explanetions are described in the revised manuscript. (Line 357- 369, page 19)

**Comment 3:** Line 435-437, incorrectly cited. The correct citation is Haile KK, Asnakew S, Waja T, Kerbih HB. Shift work sleep disorders and associated factors among nurses at federal government hospitals in Ethiopia: a cross-sectional study. BMJ Open. 2019 Aug 27;9(8):e029802

**Authors^’^ response**: **thank** **you** **reviewer** **for** **your** **comment**. We have done vigorius editing and correction in the references and the requirments for each article citation. We have revised and recited the reference mentioned with the correct citation form. (Line 164-166, page 23).
